# Supplementary material for: Cognitive and affective empathy in binge drinking during late adolescence
Source: PLoS One. 2026 Mar 10;21(3):e0341842. doi: 10.1371/journal.pone.0341842 (PMC12974825; doi:10.1371/journal.pone.0341842)
Supplement: Attachment — Submitted filename: Lester_R2R_final.pdf [file pone.0341842.s003.pdf]

Response to Reviewers

REVIEWER #1

Reviewer #1(R#1): The manuscript "Cognitive and Affective Empathy as Relating to Binge-Drinking in Late Adolescence" aims to explore the relationship between cognitive and affective empathy and binge-like consumption among college students in the United States. Although the study presents a relevant issue, some points that negatively affect the results and conclusion.

R#1: Minor concerns: the authors should standardize the terms when the abbreviation was admitted (i.e., binge drinking or BD, extreme binge drinking or EBD, etc).

AUTORS' Response: Thank you. We have changed it throughout the manuscript.

R#1: Major concerns:

- **R#1:** On page 3, the binge drinking is 0.08g percent or 0.08g/dL, not only 0.08g.

AUTORS' Response: Thank you. We have changed it accordingly (p. 3, line 27).
Binge drinking (BD) is defined as a pattern of drinking that leads to a blood alcohol concentration of 0.08 percent **- or 0.08 grams of alcohol per deciliter** - or more, generally defined as five or more standard drinks for men and four or more standard drinks for women (National Institute of Alcohol Abuse and Alcoholism [NIAAA], 2023).

- **R#1:** In the definition of adolescence age, I suggest including the WHO recommendations.

AUTORS' Response: Thank you for this suggestion. We have included it in our manuscript (p. 4, lines 60-62).
For example, the World Health Organization defines adolescence as the phase of life between childhood and adulthood, i.e., between the ages of 10-19 (WHO, 2024).

- **R#1:** On page 5, the authors affirm "Literature indicates that alcohol exposure is not associated with a general reduction in empathy overall, but rather, a dissociated pattern of empathy characterized by impaired affective empathy and preserved cognitive empathy (Maurage et al, 2011), in alcoholic adults. This pattern of preserved affective empathy and impaired cognitive empathy was also observed in adolescents (Laghi et al., 2019).". Are these sentences correct?

AUTORS' Response: Thank you for pointing it out to us. This was a typo; we have corrected it (p. 5, lines 92-93).
This pattern of ~~preserved~~ impaired affective empathy and ~~impaired~~ preserved cognitive empathy was also observed in adolescents (Laghi et al., 2019).

- **R#1:** Several terms have been modified to reduce stigma and negative bias in addiction (see https://nida.nih.gov/nidamed-medical-health-professionals/health-professions-education/words-matter-terms-to-use-avoid-when-talking-about-addiction). Thus, I suggest modifying the terms "abuse", "drinking habits", etc.

AUTORS' Response: Thank you for this important note. We have made every effort to use bias-free language; however, it appears that some terms with negative connotations have still slipped through. We have revised the manuscript to address this (e.g., replacing "abuse" with

“unhealthy alcohol use”). In other instances, the term “abuse” appears in the official name of the National Institute on Alcohol Abuse and Alcoholism, which we cannot change and must retain in citations.

- **R#1:** It is not clear the difference between extreme binge drinking vs problematic drinking.

AUTORS’ Response: Thank you for pointing out the potential for confusion. In our study, we used two methods to assess the extremity of drinking behavior in our participants. The first is categorical, consisting of three groups: non-binge drinking (non-BD), binge drinking (BD), and extreme binge drinking (EBD). The second is continuous, and is referred to as “hazardous alcohol consumption.” The former is assessed in our survey using the existing definitions of BD and EBD, and the latter is assessed in a different portion of the survey using the Alcohol Use Disorders Identification Test (AUDIT-C). This allows us to more completely examine the relationship between empathy and alcohol consumption. We can use the existing BD and EBD categorizations to examine the relationship between empathy and each level of consumption, but by including a measure of hazardous drinking, we are also able to see the relationship between empathy and alcohol consumption without the constraints of grouping to see if it exists outside of those boundaries. This approach of using both the AUDIT-C and a binge drinking assessment was also used in our foundational literature (see Lannoy, 2020). To reduce potential confusion, we have gone through to ensure consistency in the terminology used (hazardous alcohol consumption vs drinking group), and added clarifications when these terms are initially mentioned.

- **R#1:** Family drinking consumption is an important neglected variable, which may underlie adolescent behavior and consumption.

AUTORS’ Response: Thank you for pointing this out. While we did not include this in our survey, we have included a note about the importance of this variable for future research (p. 17, lines 366-370).

Another important factor to consider is familial alcohol consumption, as adolescents with a family history of alcohol misuse are more likely to exhibit problems and consequences associated with alcohol consumption [48].

- **R#1:** Why male participants were more likely to be in the EBD group than females?

AUTORS’ Response: Thank you for suggesting that we expand on this. While the fact that males are more likely than females to consume alcohol at higher levels is well established (NIAAA, 2023 & White, 2005), the field is not yet at a consensus as to why. One explanation could be a complex interplay of biological and socio-cultural effects. We have included a note about this and the implications of our findings in the discussion (p. 15, lines 312-320).

Next, our study demonstrates that men were more likely to be in the EBD group than women, which is consistent with past research indicating that men are twice as likely as women to report high-intensity alcohol use [1, 40], and are more likely than women to drink at two or three times the BD threshold [8]. This gender difference in binge drinking persists across cultures and countries, regardless of whether or not the researchers used gender-specific criteria, suggesting that this gap is not simply due to either biological or cultural differences, but rather, a complex combination of the two [40]. These findings reinforce that the motivational structure for the extremity of alcohol consumption differs between men and women across cultures, and future research should consider investigating these differences further.

- **R#1:** In my opinion, the study was not representative of the USA, since only two universities were explored. The question that remains is whether the results are cultural characteristics of Virginia state and reflect American adolescents.

AUTORS’ Response: Thank you for this note. We have added a note about the importance of this distinction for the interpretation of our paper (p. 15, lines 321-329).

Our work also highlights the prevalence of EBD within the college-aged population. The NIAAA indicates that 12% of college students engage in alcohol consumption at the EBD threshold [1], which is notably lower than the 20.69% of our sample. This discrepancy may be due to the non-representative nature of our sample, which consisted predominantly of women (73.28%) and was drawn from two universities in North Carolina, the University of North Carolina at Chapel Hill and North Carolina State University. Therefore, the nonrepresentative composition of participants should be considered as a significant limitation of this study's interpretation, and future research should aim to include participants from a more nationally representative sample.

REVIEWER #2

Reviewer #2: Thank you for the opportunity to review this study. Attached are a number of critiques I hope will improve the study. Most focus on methods, controls, consideration of patterned responses in online survey data, sample demographics and method of sampling, as well as consideration of limitations that may temper results. I also include a few articles that may help with interpretation of your data to provide a more holistic introduction to the study to more adequately represent the current science on this topic.

Overview

Authors conducted a survey of US college students (N=120) to test the association between empathy and alcohol use. The specific hypothesis being tested includes: (1) affective empathy positive associates with alcohol consumption (I believe more affective empathy relates to more drinking is the hypothesis) and (2) cognitive empathy not associating with alcohol consumption. Authors found no relationship between empathy and alcohol use in an online survey convenience sample of 18-22 aged college students that self-selected to complete the questionnaire. This is an important study but there are considerable concerns as to the reliability of the data, measurement, and analysis that lessen my enthusiasm of the study conclusions.

Conceptual issues

- **R#2:** Adolescence: The authors report this is an adolescent sample, but many would not include 18-22 in the adolescent category – rather early adulthood or emerging adulthood. Please justify this.

AUTORS' Response: Thank you for the feedback. We understand the concern regarding age classification. In our study, we adopted a broad definition of late adolescence, which includes ages up to 24 years, based on contemporary research suggesting that significant brain development, particularly within the social brain, continues well into the early twenties (Carbia et al., 2021; Sawyer et al., 2018). Therefore, for clarity and alignment with this updated understanding, we refer to individuals aged 18-24 as late adolescents throughout this paper. However, to be transparent, we have also included the WHO definition of adolescence, which does not include this age range (p. 4, lines 59-67):
The definition of the age range that adolescence encompasses has historically varied greatly depending on the context. For example, the World Health Organization defines adolescence as the phase of life between childhood and adulthood, i.e., between the ages of 10-19 [10]. However, some modern scientific research indicates that a broader definition should be considered due to changes in societal norms and new research about brain development [11, 12]. The social brain, in particular, undergoes rapid development during adolescence, which is more commonly understood in modern research to extend to 24 years old [11, 12]. As social facilitation is regarded as a key factor in adolescent drinking habits [13], in this paper, late adolescence will refer to the ages of 18-24.

- **R#2:** The assertion of "mixed" results when evidence suggests nuanced is a more appropriate characterization.

AUTORS' Response: Thank you. We have changed the wording as suggested (p. 2, line 19).
While the relationship between drinking behavior and empathy may be more nuanced than expected, ….

- **R#2:** Please see papers to consider adding below.

Methods issues

- **R#2:** No report of reliability of factor structure of measures used. I have no way of knowing how reliable these measures are because this information is not reported for the sample.

AUTORS' Response: Thank you! We have added Cronbach's alpha to the subscales. Cronbach's alpha was 0.82 for the QCAE cognitive subscale and 0.77 for the QCAE affective subscale, demonstrating, therefore, good reliability. The QCAE is considered a reliable and valid empathy measure (Reiners et al., 2011). See p. 11-12, lines 246-247.
The mean QCAE cognitive empathy score was 61.35 (SD = 6.66; Cronbach's alpha 0.82) and affective empathy 36.41 (SD = 5.22; Cronbach's alpha 0.77).

- **R#2:** No comparison of distribution of scores used in the current sample with other samples

AUTORS' Response: Thank you for this remark. We have added a note that the QCAE values of our sample are comparable to those of the normative sample (see Table 1).
The QCAE values of our sample are comparable to those of the normative sample: cognitive empathy, women M = 59.42 (SD = 0.30), men M = 56.12 (SD = 0.50); affective empathy, women M = 36.76 (SD = 0.20), and men M = 32.27 (SD = 0.30) [33].

- **R#2:** Are the self-report measures used producing a distribution different than clinical samples? What about other normative samples?

AUTORS' Response: As stated above, the QCAE values of our sample are comparable to those of the normative sample (see Table 2). To further justify the use of this measure, we included Cronbach's alpha in our QCAE score reporting, demonstrating good reliability. Moreover, the QCAE has been shown to be reliable in self-report surveys and clinically relevant (see Y. Liang, 2020). Similarly, the AUDIT has been proven reliable in both foundational literature and clinical populations, including online self-report (Simon et al., 2024). Given the literature we are building on, we believe this evidence sufficiently supports our approach and interpretations, and we included these papers to justify our methodology to readers.
See p. 9, lines 184-187.
The AUDIT-C is a reliable measure for assessing problematic alcohol consumption in adolescence [31]. It has been shown to have excellent reliability in both online and in-clinic modalities [32], and used by Lannoy et al. (2020) [28], which serves as a foundational reference for our current investigation.
See p. 9, lines 193-194.
The QCAE has been found to have both good reliability and validity in self-report surveys as well as in clinical populations [33, 34].

Controls in the model

- **R#2:** There needs to be a more thoughtful consideration of the controls used in the analysis. Specifically comorbid symptoms that impact both empathy and drinking (considering collider effects and unaccounted for variance not allowing the analysis to accurately detect signals of interest due to unaccounted for variance.
    - ADHD, externalizing, internalizing… etc.

AUTORS' Response: You are correct; more careful consideration of control variables, especially comorbid symptoms affecting both empathy and drinking, could have improved the accuracy of the results. Yet, our goal was to keep the questionnaire manageable, within a 20-minute duration, as longer surveys may introduce response bias. However, future studies

could certainly benefit from collecting and controlling for these additional variables, and we have noted this as a limitation in our study (p. 17, lines 370-377).
Additionally, research has demonstrated a notable relationship between rates of attentional deficit hyperactivity disorder and frequency of binge drinking in college students, with higher levels of inattention and hyperactivity being associated with a higher level of binge drinking [45]. Relatedly, research has also shown that adults with subclinical ADHD exhibit impaired emotional empathy [46]. As our survey did not assess ADHD or related symptoms such as inattention/hyperactivity, this presents a significant limitation to our findings and future studies should account for ADHD or related traits when examining empathy and hazardous alcohol consumption.

- o **R#2:** The authors need substantively more reporting and discussion to make the reader believe they can trust results when there does not seem to be much consideration on these controls.

AUTORS' Response: Thank you for this note. We agree that transparency and clear discussion of realistic interpretation of results are important in the reporting of research. We have gone back to add substantially more reporting and discussion regarding, specifically, the limitations of this study, the justification for our approach, reliability of our measures, the controls to consider in future research, and the interpretation of this work within the context of our experimental design. Some examples of these are described in earlier points. We believe this has greatly improved the strength of this paper.

No consideration of carelessness of responses in a survey.

- **R#2:** It is well known online surveys are prone to substantial issues in the data collected.
- **R#2:** The authors are missing a key component of working with these online surveys by not identifying highly patterned responses. These can be aided with r packages such as "careless". At this least this needs to be highlighted as a substantial limitation but highly recommend considering identifying and either controlling for or removing participants with highly patterned responses.

AUTORS' Response: Thank you for this suggestion. We are aware of the limitations associated with online surveys. Upon our initial submission, we attempted to identify careless response patterns by filtering out participants who failed the attention check or had responses more than 3 standard deviations above the mean. We have now also included additional measures of careless responses, such as calculating Mahalanobis distance. We also strengthened the deviations from the group mean for QCAE and AUDITC-C. We excluded those participants whose empathy or AUDIT-C scores deviated by more than ±2.5 SD (and not 3 SD as upon our initial submission) from the group mean. We believe these measures adequately address the concerns raised (p. 7, lines 140-147). Note that this new approach led to some small changes in the results, but the overall pattern and direction of the effects remained the same.
Further, to account for potential careless responses, we calculated Mahalanobis distance for 31 of the QCAE items using the R package "careless" [30]. Although the QCAE is a single scale, it consists of multiple subscales, allowing for the assessment of multivariate outliers using the Mahalanobis distance. Three participants who exhibited large Mahalanobis distances, indicating the presence of multivariate outliers, were excluded. Three more participants were excluded whose either empathy (cognitive or affective) or audit scores deviated by more than ±2.5 SD from the group mean.

The use of mean comparisons and simple linear regression.

- **R#2:** Simply put – there is a high level of probable bias in this analysis due to missing variable bias, sample selection, and no way to accurately assume perfect measurement (as is assumed in a linear model), therefore the results of this analysis are highly suspect and much more reporting is required to ensure I can trust the results.

- **R#2:** There needs to be a consideration of a mixed effects modeling approach to redress missing variable bias (that is highly likely) or an SEM approach that redresses measurement error (linear modeling assumes perfect measurement) when the reader has not been convinced that measurement is at least adequate for analysis.

AUTORS' Response: Thank you for your feedback. We agree that missing variable bias, sample selection, and measurement assumptions can affect analyses like this one. To address these concerns, we have already run several *preregistered* statistical models—multinomial logistic regression, ANOVA, and correlation analysis—as outlined in our preregistration. Each of these models consistently returned non-significant results.

While your point regarding mixed-effects models and SEM for handling missing variables and measurement error is valid, we believe that the consistent non-significance across various models strengthens the robustness of our findings.

Sampling

- **R#2:** The sample pool and selection process are of considerable limitation that needs to be stated explicitly.

AUTORS' Response: Thank you for this note. We did not collect information about the universities of the participants to protect their privacy. To increase transparency with readers, we noted this in our methods and have chosen to explicitly acknowledge as a limitation that the survey primarily targeted students at two universities.

p. 8, lines 156-159.

While we targeted primarily students at the University of North Carolina at Chapel Hill and North Carolina State University, we did not collect information about the universities of the participants to protect their privacy.

p. 15, lines 321-329.

Our work also highlights the prevalence of EBD within the college-aged population. The NIAAA indicates that 12% of college students engage in alcohol consumption at the EBD threshold [1], which is notably lower than the 20.69% of our sample. This discrepancy may be due to the non-representative nature of our sample, which consisted predominantly of women (73.28%) and was drawn from two universities in North Carolina, the University of North Carolina at Chapel Hill and North Carolina State University. Therefore, the nonrepresentative composition of participants should be considered as a significant limitation of this study's interpretation, and future research should aim to include participants from a more nationally representative sample.

Limitations

- **R#2:** Please consider limitations that temper the conclusions made in the study – I do not see a limitations section and no discussion on how the substantive difficulties with the sample, sampling strategy, methods used, and controls that would aid the detection of signal versus noise limits conclusions made.

AUTORS' Response: Thank you for this note. We have gone back to more transparently state the significant limitations to our study and suggestions to improve the strength of future research throughout our discussion.

R#2: Papers that need to be considered as part of the interpretation of data

R#2: https://doi.org/10.1177/00220426231159303

o This article covers substance use more broadly but investigates a clinical sample rather than highly educated and well-off college students.

- Would be a good addition to consider theoretical reasoning for indirect effects on drinking behavior and interpretation of results

AUTORS' Response: Thank you. We have incorporated this study (p. 17, lines 354-365).
Indeed, in a study conducted with a clinical adolescent population as opposed to a population of college students, higher levels of cognitive empathy predict greater response to the social consequences of alcohol consumption, implicating that a dissociation of cognitive and affective empathy may indeed exist within contexts outside of this non-representative sample **[46].** Interestingly, the participants in their study [46] were all admitted to substance use treatment and all lived in six large American cities, making it hard to conclude what variables may have contributed to our difference in findings. College students also represent a unique population to study empathy in due to the fact that scores on empathy subscale of the Interpersonal Reactivity Index have gradually decreased over time, with more recent generations demonstrating lower emotional connectivity and perspective taking [47]. This points to a need for research with diverse samples in order to better understand the motivational structure and influences underlying hazardous alcohol consumption.

R#2: https://doi.org/10.1016/j.drugalcdep.2020.108479

- o A meta-analysis showing a positive association between empathy deficits and alcohol use across studies
- o This would provide important context and help leverage evidence on alcohol and empathy.
- o The authors suggest there is mixed evidence, but the inclusion of this article leverages evidence across studies that the relationship is less "mixed" and more nuanced.
- This article also helps support the use of age and sex as controls in this study.

AUTORS' Response: Thank you. We have incorporated this into our manuscript (p. 5, lines 93-95).
Across the literature, cognitive and affective empathy are seen to be associated in some way with both current and future alcohol use severity, but with only affective constructs demonstrating a direct association [23]

R#2: Papers showing that empathy is lower in college students – suggesting that maybe not the best sample to conduct experiments on for this topic (at least needs to be listed in the limitations)

- o https://doi.org/10.3390%2Fijerph191811330

AUTORS' Response: Thank you for this suggestion. But we decided not to incorporate this content, as it focuses specifically on cognitive empathy in college students during the COVID-19 pandemic. While the findings from this study may provide valuable insights within its niche context, this paper's limited scope makes it difficult to integrate into our narrative without veering off-topic.

o **R#2:** https://doi.org/10.1177/1088868310377395

AUTORS' Response: Thank you. We have incorporated this study (p. 17, lines 360-365).
College students also represent a unique population to study empathy in due to the fact that scores on empathy subscale of the Interpersonal Reactivity Index have gradually decreased over time, with more recent generations demonstrating lower emotional connectivity and perspective taking [47]. This points to a need for research with diverse samples in order to better understand the motivational structure and influences underlying hazardous alcohol consumption.
