# Supplementary material for: Cognitive and affective empathy in binge drinking during late adolescence
Source: PLoS One. 2026 Mar 10;21(3):e0341842. doi: 10.1371/journal.pone.0341842 (PMC12974825; doi:10.1371/journal.pone.0341842)
Supplement: Attachment — Submitted filename: R2R_Lester_ep.pdf [file pone.0341842.s004.pdf]

Dear Reviewers,
We are grateful for the opportunity to resubmit our manuscript to PLOS ONE. We fully acknowledge that the original submission was at times disjointed in flow and structure. In response, we undertook a comprehensive restructuring of the manuscript, with particular focus on the introduction and discussion sections. To support this process, we invited an additional co-author to join the team (N.B.), and together with the senior author, the manuscript was carefully reworked to improve coherence, clarity, and emphasis on the key arguments.

In revising the text, we also added new references to reinforce our claims and introduce nuance, particularly in cases where the literature presents diverging findings. These additions and the structural revisions have, in our view, substantially strengthened the manuscript. Importantly, while the manuscript has been substantially restructured for clarity and coherence, the core content, results, and conclusions remain unchanged. What has changed is the organization and presentation of the research investigation, which we believe now communicates the work far more effectively. All authors have carefully reviewed and fully approved this revised version of the manuscript.

We believe this version is now well-positioned for publication.

Ekaterina Pronizius, on behalf of all co-authors

- 1. The introduction is disjointed. The paragraph describing the authors' definition of "adolescence" seems out place and interrupts the flow of their argument.
- 2. Additionally, the authors restate their study aims multiple times in the introduction (lines 31-32; 52-58; 123-128). I recommend they restructure the section and remove any redundancy.
- I recommend the authors to do a thorough copyedit of the manuscript.

Please see our response above and refer to the main manuscript for the revised introduction and discussion sections. In brief: We fully acknowledge that the original submission was at times disjointed in flow and structure. In response, we undertook a comprehensive restructuring of the manuscript, with particular focus on the introduction and discussion sections. We also conducted a thorough copyedit of the manuscript, correcting grammar, spelling, and punctuation, and standardizing abbreviations and capitalization throughout.

- 3. Some of the methods are presented in the introduction (lines 55-58).

Guided by your feedback, we relocated the AUDIT-C description to Methods and refined the Introduction's closing to improve coherence.

Methods:

- 1. Line 132 says they targeted students attending a university but in line 157-158, they name two universities. Moreover, what was the rationale for selecting these institutions?

Thank you for the comment. Our study used a convenience sample selected for ease of survey distribution. We have clarified this in text and made this designation explicit throughout the manuscript.
A convenience sample was recruited via social media, listservs, and word of mouth, targeting students enrolled at the two nearby US universities.

- 2. Line 132-133: need consistent formatting of dates

We appreciate the feedback and have revised the text to ensure consistency throughout the manuscript.
Participants were recruited between July 11th and August 1st, 2023.

- 3. Some of the study's results are reported in the methods sections, i.e. the demographic breakdown of the final sample and the number of participants excluded from the final analysis. I recommend authors move the information to the results section and/or expanding table 1 to also include the gender and racial breakdowns of the final sample

Thank you for your suggestion. We moved the demographic breakdown to the results section and extended the table with gender and racial breakdowns of the final sample.

Technical comments:

- 2. There were a few inconsistencies with abbreviations. For example: lines 32, 56

Regarding abbreviation inconsistencies (e.g., lines 32 and 56), we have corrected them. To ensure clarity, we now define it at first use as "non–binge drinking (non-BD)" and apply it consistently thereafter.

- 3. There are inconsistencies with citation styles within the paper.

We have revised all in-text citations and references to conform to PLOS ONE's Vancouver style.
