# Supplementary material for: Cognitive and affective empathy in binge drinking during late adolescence
Source: PLoS One. 2026 Mar 10;21(3):e0341842. doi: 10.1371/journal.pone.0341842 (PMC12974825; doi:10.1371/journal.pone.0341842)
Supplement: Attachment — Submitted filename: Overview.docx [file pone.0341842.s002.docx]

Overview

Authors conducted a survey of US college students (N=120) to test the association between empathy and alcohol use. The specific hypothesis being tested includes: (1) affective empathy positive associates with alcohol consumption (I believe more affective empathy relates to more drinking is the hypothesis) and (2) cognitive empathy not associating with alcohol consumption.

Authors found no relationship between empathy and alcohol use in an online survey convenience sample of 18-22 aged college students that self-selected to complete the questionnaire.

This is an important study but there are considerable concerns as to the reliability of the data, measurement, and analysis that lessen my enthusiasm of the study conclusions.

Conceptual issues

Adolescence

- The authors report this is an adolescent sample, but many would not include 18-22 in the adolescent category – rather early adulthood or emerging adulthood. Please justify this.

The assertion of “mixed” results when evidence suggests nuanced is a more appropriate characterization.

- Please see papers to consider adding below.

Methods issues

No report of reliability of factor structure of measures used.

- I have no way of knowing how reliable these measures are because this information is not reported for the sample.

No comparison of distribution of scores used in the current sample with other samples

- Are the self-report measures used producing a distribution different than clinical samples? What about other normative samples?

Controls in the model

- There needs to be a more thoughtful consideration of the controls used in the analysis. Specifically comorbid symptoms that impact both empathy and drinking (considering collider effects and unaccounted for variance not allowing the analysis to accurately detect signals of interest due to unaccounted for variance.
  - ADHD, externalizing, internalizing… etc.
  - The authors need substantively more reporting and discussion to make the reader believe they can trust results when there does not seem to be much consideration on these controls.

No consideration of carelessness of responses in a survey.

- It is well known online surveys are prone to substantial issues in the data collected.
- The authors are missing a key component of working with these online surveys by not identifying highly patterned responses. These can be aided with r packages such as “careless”. At this least this needs to be highlighted as a substantial limitation but highly recommend considering identifying and either controlling for or removing participants with highly patterned responses.

The use of mean comparisons and simple linear regression.

- Simply put – there is a high level of probable bias in this analysis due to missing variable bias, sample selection, and no way to accurately assume perfect measurement (as is assumed in a linear model), therefore the results of this analysis are highly suspect and much more reporting is required to ensure I can trust the results.
- There needs to be a consideration of a mixed effects modeling approach to redress missing variable bias (that is highly likely) or an SEM approach that redresses measurement error (linear modeling assumes perfect measurement) when the reader has not been convinced that measurement is at least adequate for analysis.

Sampling

- The sample pool and selection process are of considerable limitation that needs to be stated explicitly.

Limitations

Please consider limitations that temper the conclusions made in the study – I do not see a limitations section and no discussion on how the substantive difficulties with the sample, sampling strategy, methods used, and controls that would aid the detection of signal versus noise limits conclusions made.

Papers that need to be considered as part of the interpretation of data

- <https://doi.org/10.1177/00220426231159303>
  - This article covers substance use more broadly but investigates a clinical sample rather than highly educated and well-off college students.
  - Would be a good addition to consider theoretical reasoning for indirect effects on drinking behavior and interpretation of results
- <https://doi.org/10.1016/j.drugalcdep.2020.108479>
  - A meta-analysis showing a positive association between empathy deficits and alcohol use across studies
  - This would provide important context and help leverage evidence on alcohol and empathy.
  - The authors suggest there is mixed evidence, but the inclusion of this article leverages evidence across studies that the relationship is less “mixed” and more nuanced.
  - This article also helps support the use of age and sex as controls in this study.
- Papers showing that empathy is lower in college students – suggesting that maybe not the best sample to conduct experiments on for this topic (at least needs to be listed in the limitations)
  - <https://doi.org/10.3390%2Fijerph191811330>
  - <https://doi.org/10.1177/1088868310377395>
